# Subjective employment perspective among older workers with and without migrant background in Germany—Results of the lidA cohort study

**DOI:** 10.1002/1348-9585.12166

**Published:** 2020-09-09

**Authors:** Chloé Charlotte Schröder, Hans Martin Hasselhorn, Jean‐Baptist du Prel, Jürgen Breckenkamp

**Affiliations:** ^1^ Department of Occupational Health Science University of Wuppertal Wuppertal Germany; ^2^ Department of Epidemiology & International Public Health School of Public Health Bielefeld University Bielefeld Germany

**Keywords:** emigrants and immigrants, employee participation, finances, motivation, older workers, retirement

## Abstract

**Objectives:**

The aim of this study was to investigate the subjective employment perspective in higher working age for different employee groups with migrant background (EMB) and without (non‐EMB), meaning willing, being able, and planning to work until the individual state pension age (iSPA).

**Methods:**

A representative sample of socially insured employees born in 1959 or 1965 was surveyed in 2011, 2014, and 2018 with computer‐assisted personal interviews. The current cross‐sectional analysis is based on data from the third study wave (n = 3286) of the lidA cohort study. EMB were differentiated via generation (first generation, G1, vs second generation, G2) or nationality (German vs foreign). Applying bivariate statistics with the tests of independence and block‐wise logistic regressions, group differences were investigated. Sex, age, educational level, net household income, health, and work factors were considered as covariates.

**Results:**

When comparing subgroups of EMB, significant differences appeared in bivariate analyses for willing and planning to work. G1 were to a higher degree planning to work longer than G2 and those with foreign nationality were more willing and planning than those with German nationality. Multivariate analyses revealed significant differences of G1 and non‐EMB for planning, being significant in the fully adjusted model, but not for willing.

**Conclusion:**

The findings underline the need for differentiation of migrant groups in social research and policy. When it comes to extended working lives, the first‐generation migrant group, as well as foreigners may constitute risk groups and require increased attention from a work, health, and economic point of view.

## 1 INTRODUCTION

In many European welfare states the extension of working lives (EWL) is regarded as an instrument to maintain wealth and social stability in times of population aging. Thus, many European states have reduced pathways and incentives for early exit from work and raised official pension entitlement age.^1^ Retirement research has addressed the issue of extending working lives by investigating determinants for early or late exit from employment and by identifying typical pathways from work to retirement. One group, however, has been virtually ignored by retirement research: those with migrant background.^2^

In general, migrants are a highly heterogeneous and diverse group with regard to their origin, culture, religion, and education.^3^, ^4^ On average, migrants may be assumed to be more vulnerable, compared to nonmigrants, from a social, employment, and economic perspective.^2^, ^5^ Regarding health status in migrants, it cannot be said that they are more or less healthy in general than nonmigrants. Findings are not consistent, as different definitions to identify migrants are used and migrant subgroups may differ in this respect. Additionally, observations substantially depend on the health outcome chosen.^6^ It was observed that migrants usually are healthier than nonmigrants, resulting in better health and lower mortality in the target country (“healthy migrant effect”). However, this finding is mostly based on the fact that usually healthier people emigrate. With increased duration of stay, the observed benefits in terms of health are gradually reduced, partly due to adaption of health‐related behavior and social status in the host country.^6^

In Germany, the proportion of employees with migrant background (EMB) is continuously growing, for example from 16.2% in 2010 to 23.9% in 2018.^7^, ^8^ The largest proportion of persons with migrant background are resettlers with German ancestry from Eastern Europe and the former Soviet Union, as well as persons of Turkish and Polish origin.^8^ They are overrepresented in jobs affected by economic restructuring, such as manual and un/semi‐skilled blue‐collar work.^8^, ^9^, ^10^, ^11^ Employees with foreign nationality more frequently suffer from occupational accidents and diseases, as well as retire earlier in the form of disability pension, compared to employees with German nationality.^10^, ^12^ Such health risks may be attributed to more physically demanding work, but also to lower utilization of health services.^5^, ^12^, ^13^

In the coming years, large groups of EMB will reach pensionable age. In 2018, 3.7 million EMB were 45 to 64 years old. This accounted for 17.9% of all workers of that age group and 37.3% of all 10 million EMB in Germany.^8^ Older EMB mainly work in blue‐collar positions (55%), 32% in white‐collar occupations, about 13% are sworn civil servants or self‐employed; this pattern is found for all age groups in EMB. The average monthly net income of EMB is 1965 € compared to 2470 € among employees without migrant background (non‐EMB).^8^

What generalized statements do not reflect, however, is the substantial variation within the group of EMB. In a recent empirically based summary report, the European Agency Eurofound records substantial differences in working conditions between EMB of first‐ and second generation and calls for differentiation between these groups in policymaking.^14^ The first generation (G1 EMB) is born in another country than the host country, whereas the second generation (G2 EMB) has one or two parents who are foreign‐born, but no own migration experience. Hence, Eurofound and others suggest that G2 EMB may be more similar to non‐EMB due to adaptation processes while growing up in the host country, than G1 EMB.^14^ In German representative surveys, older G1 EMB were found more frequently in unskilled blue‐collar positions than older G2 EMB,^8^, ^11^ and to a higher extent exposed to adverse work factors with increased health risks, such as adverse work postures and low influence at work.^11^ Mean monthly net income was lower (1904 €) for G1 EMB than for G2 EMB (2630 €), which is even higher than for non‐EMB (see above).^8^

Such group differences may be of relevance for policymaking and organizations in the context of EWL, when interventions aimed to promote work participation are considered. Concerns have been brought forward that current EWL policies relocate additional health and social risks to different groups of older EMB.^15^ The subjective employment perspective of EMB and non‐EMB discussed here may represent an early indicator of employment participation in the coming years. A crucial question will be whether the subjective employment perspective of the G2 EMB resembles that of G1 EMB or non‐EMB.

In our study, the subjective employment perspective is operationalized by willing, able, and planning to work until a certain age, to capture a range of indicators. Conceptual differences between these three outcomes may be assumed.^16^, ^17^ Planning as expected and willing as preferred retirement age were found to be good indicators for future retirement behavior.^18^, ^19^ The employment perspective is influenced by a wide range of factors during the process of retirement, such as personal factors and social and occupational contexts.^18^, ^20^ It was found that older employees in precarious job positions with low education and income, where for example G1 EMB might be part of, would like to retire earlier than they plan to, probably due to financial reasons.^20^, ^21^ The aspect of able to work is likewise essential, as EMB might not be able to work as long as they plan to or should, because of their working conditions which affect their health status.^16^

Research on the work‐retirement transition considering migrant subgroups is rare. In a German study of older employees, G1 EMB were found to be significantly less willing to retire before the age of 65.^20^ A Canadian study found that immigrants (first generation) planned to retire later than native Canadians.^22^ Representative studies systematically investigating the employment perspective in older employees with distinct differentiation of migrant background are largely lacking. Large quantitative studies on migrants’ work, health, and/or employment in Germany often suffer from severe limitations when they are based on secondary data.^10^, ^23^ In most such datasets, migrant background is solely indicated by “nationality,” thus not permitting a differentiation of migrant background and misclassifying about half of all people with migrant background as nonmigrants; as 9.7 million, of a total 19.6 million people with migrant background, were Germans in 2018.^8^

Instead, the third wave of the lidA study allows for differentiating distinct groups with migrant background among older workers and relating them to different aspects of the subjective employment perspective.

### 1.1 Research question

The aim of this study was to investigate the subjective employment perspective in higher working age for different groups with and without migrant background, meaning willing, able to, and planning to work until the individual state pension age (iSPA). Group differences should be determined and the impact of sociodemographic, health, and work factors should be investigated.

## 2 METHODS

### 2.1 Study design and participants

The lidA (leben in der Arbeit) cohort study investigates work, health, and work participation in the older workforce in Germany. This study examines a representative sample of older employees, who were born in 1959 or 1965 and socially insured during sampling in 2009. Due to this sampling specification, sworn civil servants and self‐employed were not included. The participants were interviewed at home for each assessment wave by computer‐assisted personal interviews (CAPI), covering topics such as work, health, private life, and employment perspective. The baseline survey took place in 2011 (n = 6585), the second wave in 2014 (n = 4244), and the third wave in 2018 (n = 3586). A detailed description of the lidA cohort study and its sampling process can be found elsewhere.^24^ The lidA datasets of the first and second wave are available as a Scientific Use File,^25^ data from the third wave will be added by 2023.

Results of attrition analysis showed for all waves a widely selection‐free realization of the sample in relation to the sociodemographic characteristics used in the analyses.^26^, ^27^, ^28^ However, attrition from the first to the third wave was 47% for the total sample, for low educational level it was 76% in G1 EMB compared to about 53% in non‐EMB and G2 EMB. Since this report is based on data from the third study wave, we performed inverse probability weighting for subgroups of migrant status and educational level.

The sample was restricted to those being employed at least 1h/week. Due to the weighting, cases with missing values in migrant background or educational level were excluded as well. Consequently, the final sample consisted of 3286 individuals.

### 2.2 Operationalization

#### 2.2.1 Outcomes

The main outcome of the analysis is the subjective employment perspective which was parameterized by three single outcomes: *willing, able,* and *planning* to work until the individual state pension age (iSPA). Participants were asked until what age they would like (*willing*), they think they would be able to (*able*), and they plan to work (*planning*). Responses given had to be *years of age*. For the analyses in this study, the answers were dichotomized into less than vs at least until the current iSPA in Germany, which is 66 years of age for the 1959‐cohort and 67 years of age for the 1965‐cohort. The outcomes have only been surveyed in such detail in the third wave so far.

#### 2.2.2 Migrant background

The lidA cohort study allows to distinguish between migrant groups by means of different specific indicators as proposed by Schenk et al.^29^ EMB were defined based on the participants’ self‐reported country of birth and nationality and on the country of birth of each of their parents. Participants born in Germany, with German citizenship and with both parents being born in Germany constitute the reference group (non‐EMB). To investigate the group of EMB, two different operationalizations were adopted: The first operationalization is based on a definition provided by the German Federal Statistical Office,^7^, ^8^ where EMB are separated by generation into first generation (G1 EMB) and second generation (G2 EMB), as described before. For some analyses, the group of G2 EMB was further separated into participants with one or two parents born outside Germany to investigate potential differences between unilateral and bilateral foreign descent. The second operationalization of EMB is based on nationality (German/dual vs foreign) to reflect the more detailed differentiation level in contrast to process data as indicated above.

#### 2.2.3 Covariates

As the three outcome variables might be influenced by further factors besides the migrant background,^2^ the following variables were considered as potential confounders. Year of birth (1959/1965), sex (male/female), education, and financial situation comprise the sociodemographic factors. Education was parameterized with the help of a score combining educational and vocational training and then classified in three categories from high to low level.^30^ To measure the financial situation, the net equivalent household income was used. It represents the mean net income of each person in a household weighted for the number and age of the persons living in the household. The square root scale of the OECD was applied.^31^ The net income variable was grouped into categories of < 60%, <80%, <100%, <150%, and >150% of the sample median (2249.75€), where <60% may indicate risk of poverty.^32^

Physical and mental health were considered as further covariates. These were assessed by two established scales of the SF‐12 Health Survey^33^ in an adapted German version.^34^ The physical component summary scale (PCS‐12) considers physical health and the mental component summary scale (MCS‐12) considers the respondents’ mental health; higher scores indicate better health.

Further control variables were added to adjust for different occupational exposures of potential relevance for the outcomes of interest.^18^, ^20^, ^35^ Influence at work was assessed with three items (influence on with whom, what, and how much one works, COPSOQ II, middle version), with a mean ranging from 0 (no influence) to 100 (high influence).^36^ Work‐related stress was assessed with the effort‐reward imbalance (ERI) scale which was used as a continuous measure. Imbalance was measured with the ERI ratio, the quotient of the effort and the reward scale by adding a weighting factor to adjust for the different numbers of items in the nominator and denominator. Values close to minimum of 0.2 express low work stress while values above 1.0 indicate a very high ERI imbalance, meaning higher personal work stress.^37^

For physical work load, a cumulative measure was drawn up from exposure to adverse postures, heavy lifting or carrying and one‐sided movements at work. The answer categories corresponded to proportions of working time (never, up to one quarter, up to half, up to three quarters, (almost) always). Any exposure greater than "never" was counted as one "exposure" in total.^38^

### 2.3 Statistical analysis

Due to group differences in attrition between the first and the third study wave, inverse probability weighting was done for subgroups of migrant status and educational level. All reported results are based on weighted analyses; however, in Table 1 additionally unweighted characteristics are presented for comparison. Descriptive and bivariate statistics including chi‐squared and Kruskal‐Wallis tests, as well as ANOVAs were used to characterize the full sample and specifically investigate differences between groups and the outcomes. To investigate potential differences between EMB and non‐EMB, multivariate logistic regressions were performed while adjusting block‐wise for sociodemographic, health, and work factors. For migrant background as the main independent variable, differentiation by migrant generation was chosen. Regressions were performed for each outcome, respectively, using complete‐case analysis. In addition, average marginal effects (AMEs) were computed for all logistic regressions with SAS 9.4. They allow us to compare the results of nested models that otherwise may be biased by unobserved heterogeneity. The AME shows for each variable in a regression model how much the event probability changes when the independent variable increases by one unit, or rather when a binary independent variable changes its level.^39^

**TABLE 1:** Characterization of study population, weighted and unweighted sample

	Weighted sample^d^ (n = 3286)				Unweighted sample (n = 3324)			
	Non‐EMB (n = 2703)	G1 EMB (n = 346)	G2 EMB (n = 236)	*P*‐value^a^	Non‐EMB (n = 2828)	G1 EMB (n = 244)	G2 EMB (n = 252)	*P*‐value^a^
Sex [n (%)]								
Male	1228 (45.4)	184 (53.2)	105 (44.5)	.021	1274 (45.0)	125 (51.2)	112 (44.4)	.168
Female	1475 (54.6)	162 (46.8)	131 (55.5)		1554 (55.0)	119 (48.8)	140 (55.6)	
Year of birth [n (%)]								
1959	1214 (44.9)	155 (44.7)	94 (39.7)	.296	1265 (44.7)	110 (45.1)	101 (40.1)	.354
1965	1489 (55.1)	192 (55.3)	143 (60.3)		1563 (55.3)	134 (54.9)	151 (59.9)	
Combined education level [n (%)]								
High	551 (20.4)	74 (21.3)	51 (21.6)	<.001	620 (22.0)	70 (30.0)	60 (23.8)	.003
Medium	1505 (55.7)	148 (42.7)	118 (50.0)		1627 (57.7)	106 (45.5)	131 (52.0)	
Low	647 (23.9)	125 (36.0)	67 (28.4)		572 (20.3)	57 (24.5)	61 (24.2)	
Net household income [n (%)], m = 119								
>150% (>3374.00€)	303 (11.6)	15 (4.5)	32 (14.0)	<.001	328 (12.1)	11 (4.7)	35 (14.3)	<.001
<150% (2249.80€‐3373.90€)	859 (33.0)	85 (25.3)	78 (34.1)		909 (33.4)	62 (26.3)	85 (34.8)	
<100% (1799.60€‐2249.75€)	558 (21.4)	58 (17.3)	49 (21.4)		581 (21.4)	42 (17.8)	51 (20.9)	
<80% (1799.50€‐1350.00€)	577 (22.2)	97 (28.9)	45 (19.7)		596 (21.9)	66 (28.0)	46 (18.9)	
<60% (<1349.90€)	305 (11.7)	81 (24.1)	25 (10.9)		306 (11.3)	55 (23.3)	27 (11.1)	
SF‐12: physical health [M (SD)], m = 11	48.1 (9.2)	46.2 (9.4)	46.9 (8.5)	<.004^b^	48.2 (9.2)	46.6 (9.4)	47.1 (8.4)	.008^b^
SF‐12: mental health [M (SD)], m = 11	51.8 (9.8)	51.4 (10.4)	52.2 (9.3)	.248^b^	51.7 (9.9)	50.7 (10.3)	51.9 (9.4)	.243^b^
COPSOQ: Influence at work [M (SD)], m = 3	37.4 (26.2)	32.9 (27.0)	37.9 (26.2)	.002^b^	37.5 (26.1)	34.0 (26.5)	37.8 (25.8)	.129^b^
Work stress, ERI [Mdn (IQR)], m = 24	0.50 (0.38)	0.42 (0.33)	0.50 (0.40)	<.001^c^	0.50 (0.38)	0.42 (0.30)	0.50 (0.39)	.004^c^
Cumulative physical work exposure								
No physical exposure	556 (20.6)	50 (14.5)	49 (20.7)	.001	597 (21.1)	37 (15.2)	54 (21.4)	.03
One exposure	963 (35.6)	102 (29.5)	73 (30.8)		1019 (36.0)	76 (31.1)	80 (31.7)	
Two exposures	586 (21.7)	105 (30.3)	52 (21.9)		607 (21.5)	69 (28.3)	55 (21.8)	
Three exposures	598 (22.1)	89 (25.7)	63 (26.6)		605 (21.4)	62 (25.4)	63 (25.0)	

Note

Bold print indicates significance, *P* < .05.

Abbreviation: EMB, employees with migrant background; G1, first generation; G2, second generation; IQR, Interquartile range; M, Mean; Mdn, Median; m, number of missing values due to respondents not responding to the item, from weighted results; SD, Standard deviation.

^a^ Tested with chi‐squared test if not otherwise specified.

^b^ Tested with ANOVA.

^c^ Tested with Kruskal‐Wallis test.

^d^ Weighting factors: for non‐EMB/low 1.134, for non‐EMB/medium 0.927, for non‐EMB/high 0.896, for G1 EMB/low 2.229, for G1 EMB/medium 1.438, for G1 EMB/high 1.081, for G2 EMB/low 1.101, for G2 EMB/medium 0.907, for G2 EMB/high 0.869.

In all statistical tests *P*‐values (two‐tailed) <.05 were considered to be statistically significant. Within the logistic regressions Nagelkerke's pseudo‐*R* ^2^ was used as a measure for comparing competing models. All statistical analyses (other than AMEs) were performed using SPSS version 25.0 (IBM Corp.).

## 3 RESULTS

### 3.1 Descriptive and bivariate analysis

Baseline characteristics of all participants included in the analyses are given in Table 1, shown as weighted (n = 3286) and unweighted results (n = 3224). Due to deliberate oversampling, participants born in 1965 were overrepresented in all subgroups. The following summary of findings refers to significant and weighted results only. The proportion of men was higher in G1 EMB than in non‐EMB or G2 EMB. The distribution of educational level differed between the three groups (*P *=< .001); the proportion of workers with low educational level was highest among G1 EMB (36.0%) compared to non‐EMB or G2 EMB. But there were no significant group differences when comparing non‐EMB with G2 EMB only (*P* = .202, chi‐squared test, not shown). The distribution of income groups differed between the three groups (*P *=< .001), yet it was rather similar for non‐EMB and G2 EMB. In G1 EMB, 53% had a household income below 80% of the median vs 34% in non‐EMB and 31% in G2 EMB. The mean score for physical health was lowest for G1 EMB (46.2), followed by G2 EMB (46.9) and non‐EMB (48.1, *P* = .004). G1 EMB had lower influence on their own work (32.9) than G2 EMB (37.9) and non‐EMB (37.4, *P* = .002). However, concerning work stress, G1 EMB had lower work stress with a median of the ERI ratio of 0.42 and 0.5 for the other two groups (*P*=<0.001**)**. Among the G1 EMB, 85% experienced at least one adverse physical exposure at work compared to 79% among the non‐EMB and G2 EMB, respectively, *P* = .001).

Table 2 displays the outcomes by different subgroups. There were no significant group differences between non‐EMB and EMB with respect to willing, able, and planning to work until one's iSPA. When comparing EMB subgroups, G1 were to a higher degree planning to work longer than G2 (25% vs 16%) and those with foreign nationality were more willing and planning than those with German nationality (18% vs 9% for willing, 29% vs 20% for planning). No differences, however, were found for able to work until iSPA. The subdivision of the EMB G2 group into those with one or two parents of foreign origin did not indicate any significant differences between the two groups.

**TABLE 2:** Willing, able, and planning to work until individual state pension age by migrant status (n = 3286), weighted results

	Willing			Able			Planning			n
	%	(95% CI)	*P*‐value^a^	%	(95% CI)	*P*‐value^a^	%	(95% CI)	*P*‐value^a^	
All EMB vs Non‐EMB										
Non‐EMB	10	(9‐11)	.497	32	(30‐33)	.097	21	(19‐22)	.706	2703
EMB	11	(8‐13)		28	(24‐32)		22	(18‐25)		583
EMB by generation										
First generation (G1)	12	(09‐16)	.128	27	(22‐31)	.387	25	(20‐30)	.014	346
Second generation (G2)	8	(5‐12)		30	(24‐36)		16	(12‐21)		236
EMB by nationality										
German or dual	9	(6‐12)	.004	28	(23‐32)	.564	20	(16‐23)	.035	464
Foreign	18	(11‐25)		30	(22‐39)		29	(20‐37)		119
EMB G2 by foreign descent										
Unilateral	9	(5‐13)	.892	29	(22‐35)	.399	17	(11‐22)	.677	187
Bilateral	7	(0‐15)		35	(21‐49)		15	(5‐25)		50

Note

Bold print indicates significance, *P* < .05.

Abbreviation: EMB, employees with migrant background; G1, first generation; G2, second generation.

^a^ Tested with chi‐squared test.

### 3.2 Multivariate analysis for willing and planning

Throughout all models, G1 EMB exhibited higher and G2 EMB exhibited somewhat lower odds ratios (OR) for willing to work until iSPA than non‐EMB. Nevertheless, these group differences were not significant, although when adjusting for health factors in model 3, the *P*‐value for G1 EMB was closely above .05.

With respect to planning to work until iSPA (Table 3), significantly higher OR were found for G1 EMB than for non‐EMB in the null model (OR = 1.34, 95%‐CI 1.03‐1.74). The probability for planning to work until iSPA was increased by 3.9%‐points in G1 EMB. Adjusting for sex, age, physical and mental health even further increased significance as well as the probability up to 5.3%‐points. When additionally considering further covariates in models 3 and 4, the probabilities and odds ratios declined, but were still significant. Between G2 EMB and non‐EMB, no significant differences were found in any model. Respective findings for the outcome able to work were not shown or discussed as there were no statistically significant group differences.

**TABLE 3:** Association for willing and planning to work until the individual state pension age with migrant status, weighted results

	Model 0:	Model 1:	Model 2:	Model 3:	Model 4:
	Crude	M0 + sex, age	M1 + health	M2 + education, net household income	M3 + physical work exposure, work stress, influence at work
**Willing (n = 3135/ n_events_ = 314)**					
OR (95% CI)					
Non‐EMB	Ref.	Ref.	Ref.	Ref.	Ref.
G1 EMB	1.30 (0.91‐1.85)	1.30 (0.91‐1.86)	1.42 (0.99‐2.04)	1.29 (0.90‐1.86)	1.26 (0.87‐1.82)
G2 EMB	0.82 (0.51‐1.35)	0.84 (0.51‐1.37)	0.85 (0.52‐1.40)	0.87 (0.53‐1.43)	0.88 (0.53‐1.45)
AME					
Non‐EMB	Ref.	Ref.	Ref.	Ref.	Ref.
G1 EMB	+0.0236	+0.0234	+0.0332	+0.0208	+0.0199
G2 EMB	−0.0157	−0.0139	−0.0108	−0.0077	−0.0115
R^2^	0.002	0.008	0.035	0.063	0.084
**Planning (n = 3132/ n_events_ = 662)**					
OR (95% CI)					
Non‐EMB	Ref.	Ref.	Ref.	Ref.	Ref.
G1 EMB	**1.34 (1.03‐1.74)** *	**1.35 (1.03‐1.76)** *	**1.42 (1.09‐1.86)** **	**1.38 (1.04‐1.82)** *	**1.38 (1.04‐1.82)** *
G2 EMB	0.76 (0.53‐1.09)	0.76 **(**0.53‐1.09)	0.76 (0.53‐1.10)	0.77 (0.54‐1.12)	0.78 (0.54‐1.13)
AME					
Non‐EMB	Ref.	Ref.	Ref.	Ref.	Ref.
G1 EMB	+0.0392	+0.0397	+0.0526	+0.0488	+0.0513
G2 EMB	−0.0473	−0.0487	−0.0450	−0.0425	−0.0392
R^2^	0.004	0.006	0.018	0.071	0.074

Note

Bold print indicates significance.

Abbreviation: AME, average marginal effects; CI, confidence interval; M, Model; n_events,_ number of events where the outcome = 1 in the logistic regression; OR, Odds Ratio; *P*, *P*‐value; Ref., Reference; R^2^, Nagelkerke pseudo‐R^2^.

* *P* < .05,

** *P* < .01,

*** *P* < .001.

Secondary findings within multivariate analyses indicated that the following covariates were significantly associated with willing and planning to work until iSPA (data not shown): Belonging to the 1959‐cohort was associated with higher OR for willing, while having less than 60% mean net household income showed higher OR (around 2) for both outcomes, willing and planning. In contrast, significantly lower OR for willing and planning were found for those with medium and low educational level. Also, the ERI ratio was significantly associated with willing to work until iSPA in the expected direction.

## 4 DISCUSSION

In the present study, we analyzed the subjective employment perspective in higher working age for different groups of EMB and non‐EMB, meaning willing, able to, and planning to work until the individual state pension age. For “able to work” no group differences were found. When comparing all EMB with non‐EMB in bivariate analyses, no significant differences were observed for any of the three outcomes. However, when comparing migrant subgroups, significant differences appeared for willing and planning to work until iSPA. Among EMB, those with foreign nationality were, to a higher degree, willing and planning to work until iSPA than those of German nationality. Likewise, G1 EMB were more planning to work until iSPA than G2 EMB. Multivariate analyses revealed significantly higher odds ratios for planning among G1 EMB compared to non‐EMB, even when considering potential confounders, while there were no significant group differences for willing.

In all groups considered in the analyses, the proportion of those “able” to work until iSPA was clearly higher than that of planning and finally, followed by willing. This is in line with findings from Sweden, where 54% of the workers aged 55‐64 years stated that they “can” and 38% that they “want to” work until age 65 years or beyond.^16^ The low prevalence for planning and willing is indicative of an “early exit culture” still prevailing in Germany.^40^ The absence of significant group differences for able may reflect an even distribution of the individuals’ perception of their mental and physical resources between the groups. However, the larger group differences found for willing and especially planning may be indicative of migrant status differences with respect to the older workers’ subjective valuation of their last period of working life. The relatively high prevalence for willing among older workers with foreign nationality might thereby express a higher pressure felt to retire late among those financially less well off.^15^ In Germany, workers of foreign nationality more frequently work in un/semi‐skilled positions^11^ and consequently, have lower income than German EMB (own results, data not shown). Hess^21^ has found among older workers in Germany that financial needs were associated with a higher expected retirement age. As “expected retirement age” and “planned retirement age” may be conceptually closely related, one may conclude that finances might also contribute to the higher prevalence for planning found among non‐German EMB and G1 EMB. However, the significantly higher OR for G1 EMB for planning throughout all multivariate logistic regression models also imply inherent or further migrant status group differences. It is noticeable that in our study, the results for G2 EMB are more similar to non‐EMB than to G1 EMB. This may indicate a high degree of social integration of this generation.

The scientific literature on migrant status and employment perspective and behavior, respectively, is scarce. We are not aware of any further study investigating the outcome “planning” among older workers with respect to migrant background. In the only study known to us, investigating the outcome willing among older employees, it was found that G1 EMB were significantly more willing to work longer than non‐EMB.^20^ This analysis was based on the same sample as ours, but on an earlier study wave, which did not provide the possibility to compare the effects of the three outcomes of the employment perspective. Canadian research has identified that immigrants (first generation) intended to retire later than natives, which is in line with our findings. Concerning actual retirement, immigrants were found to be less likely to leave work early, except for involuntary early retirement such as disability pension due to poor health, which immigrants were more likely to receive than nonimmigrants.^22^ In an earlier German study, it was observed that migrants retired significantly later compared to West‐Germans when controlling for employment status at the age of 50 years and before retirement entry.^41^ However, the operationalization of the migrant status was not mentioned in the study.

By considering migrant background when investigating the work‐retirement transition, our study contributes to filling in the research gap, addressed in earlier reviews.^2^ The findings confirm the necessity emphasized by Eurofound^14^ to differentiate between migrant subgroups in the work force, as different subgroups do not experience the same problems in daily life and behave differently. One conclusion of their research was that policy should consider distinct approaches to meet the needs of different migrant subgroups. Unlike most other German datasets, the lidA cohort study has the potential to identify different migrant groups based on several indicators and not only by nationality, so that recommendations for mapping of migrant status could be followed.^29^

In retirement research, health is considered as a key determinant of early retirement.^42^, ^43^ In line with this, physical and mental health were found to be important factors in our analyses, influencing whether older employees are willing and planning to work until iSPA. When controlling for these aspects in multivariate analyses, effect estimates for G1 EMB increased for both willing and for planning to work until iSPA. This indicates that among older employees, G1 EMB were more likely to plan to work longer if they had a comparably good health status as non‐EBM (cf. Table 1 for physical health).

Further important factors in our study seemed to be the household income and the educational level, as effect estimates were somewhat decreasing for G1 and G2 EMB when additionally adjusting for these two factors. However, concerning planning, significant differences remained after full adjustment. In previous research about these sociodemographic characteristics, EMB were found to be at a higher risk of becoming unemployed or having low‐paid employment positions. During recruitment of so called “guest workers” to Germany from the late 1950s to the early 1970s, employees with low education and qualification were predominantly recruited, indicating lower income levels for G1 EMB in later life.^44^ Likewise, in our weighted sample, a comparably high percentage of G1 EMB indicated lower education and reported poorer working conditions, such as physical exposures and lower income, than non‐EMB.

Overall, not only migrants with foreign nationality, but also G1 EMB might constitute a special group to focus upon for the coming years until retirement in research and policy. In our weighted sample, this group has, on average, a lower educational level, lower household income, poor physical health, higher physical work exposures, but nonetheless reports fairly low work stress. Our findings indicate that in Germany in times of extending working lives, certain migrant groups approaching retirement age might constitute risk groups locked in lower working positions, poor health and economics where the “planning” does not reflect a choice, but a forced decision to work longer. To offset negative effects of extended working lives expected for vulnerable groups of older workers, scientists increasingly call for an improvement of work quality, job security, and also the promotion of lifelong learning as preconditions for policies aimed at extending working lives.^15^ Phillipson^45^ proposed work and retirement policies acknowledging the processes of cumulative advantage and disadvantage operating over the life course.

This study has several strengths. First, the use of a sample being representative for the socially insured employees of the considered two age cohorts. Socially insured employees cover about 80% of the German working population.^26^ Second, the lidA cohort study has the potential to distinguish between different migrant groups.^29^ Additionally, the lidA study questionnaire from the third wave allows for the differentiated assessment of the employment perspective. Another strength of this study is the consideration of different confounding sociodemographic, health, and work variables that may disguise differences in the outcomes between the investigated groups.

However, the present study also has limitations. The study design was cross‐sectional and it remains an open question to what extent the willing, able, and planning to work until a certain age might be stable until retirement, not least in times of extended working life policies. Concerning migrant status, we were not able to differentiate further relevant migrant groups, such as labor migrants vs resettlers vs refugees. An additional limitation is a potential bias into participant selection, as the study was conducted in German and therefore EMB could potentially be excluded due to language problems. However, we assumed for these participants certain German language skills when working in socially insured positions. In addition, the lidA cohort study uses two birth cohorts and is sampled within socially insured employees, which excludes sworn civil servants and self‐employed. As a result, the findings and conclusions drawn are limited to this group of older employees, only. Finally, the percentage of employees of G1 EMB was considerably lower than that in the first study wave. The latter could indicate a healthy worker survivor effect and selection bias, as individuals might have left the workforce due to poorer health and/or precarious job positions. However, we used inverse probability weighting to adjust panel attrition in migrant groups and educational levels.

## 5 CONCLUSION

Our findings underline the need for differentiation of migrant groups in social research and policy. When it comes to extended working lives, the first‐generation migrant group and foreigners may constitute risk groups and require increased attention from a work, health, and economic point of view.

## DISCLOSURES

*Approval of the research protocol:* Design and conduct of the lidA study have been approved by the Ethics Committee of the University of Wuppertal dated from 05/12/2008 and 20/11/2017.

*Informed consent:* Participants were fully informed about the aim and procedure of this study prior to giving consent to participate in this study. All procedures performed in studies involving human participants were in accordance with the ethical standards of the institutional and/or national research committee and with the 1964 Helsinki declaration and its later amendments or comparable ethical standards.

*Registry and the registration no. of the study/trial:* N/A.

*Animal studies:* N/A.

*Conflict of interest:* The authors declare that they have no competing interests.

## AUTHOR CONTRIBUTIONS

HMH had the idea for the study and developed the study design with CCS. CCS performed the analyses and wrote the first draft of the article. JB, HMH, and JP contributed with their expertise. All the authors critically reviewed and approved the final manuscript.

## References

[1] OECD . Pensions at a Glance 2017: OECD and G20 Indicators. Paris: OECD Publishing; 2017 10.1787/pension_glance-2017-en. Accessed May 6 2020

[2] Hasselhorn HM , Apt W , eds. Understanding employment participation of older workers: Creating a knowledge base for future labour market challenges [Research report]. Berlin: Federal Ministry of Labour and Social Affairs (BMAS) and Federal Institute for Occupational Safety and Health (BAuA) ; 2015.

[3] Schenk L . Migration und Gesundheit—Entwicklung eines Erklärungs‐ und Analysemodells für epidemiologische Studien. Int J Public Health. 2007;52(2):87‐96. 10.1007/s00038-007-6002-4 18704287

[4] Nowossadeck S , Klaus D , Gordo LR , Vogel C . Migrantinnen und Migranten in der zweiten Lebenshälfte. Report Altersdaten 02/2017. Berlin: Deutsches Zentrum für Altersfragen https://www.dza.de/fileadmin/dza/pdf/Report_Altersdaten_Heft_2_2017.pdf. Accessed May 6, 2020.

[5] Razum O , Meesmann U , Bredehorst M , et al. Migration und Gesundheit. Schwerpunktbericht der Gesundheitsberichtserstattung des Bundes. Berlin: Robert Koch‐Institut https://www.rki.de/DE/Content/Gesundheitsmonitoring/Gesundheitsberichterstattung/GBEDownloadsT/migration.pdf?__blob=publicationFile. Accessed May 6, 2020.

[6] Robert Koch‐Institut , ed. Gesundheit in Deutschland . Gesundheitsberichterstattung des Bundes ‐ Gemeinsam getragen von RKI und Destatis. Berlin: Robert Koch‐Institut; 2015 http://www.gbe‐bund.de/pdf/GESBER2015.pdf. Accessed May 6, 2020.

[7] Statistisches Bundesamt , ed. Bevölkerung mit Migrationshintergrund: Ergebnisse des Mikrozensus 2010 ‐ hochgerechnet auf Basis des Zensus 2011. Fachserie 1 Reihe 2.2. 2017 https://www.destatis.de/DE/Themen/Gesellschaft‐Umwelt/Bevoelkerung/Migration‐Integration/Publikationen/Downloads‐Migration/migrationshintergrund‐sonderausgabe‐5122121109004.pdf?__blob=publicationFile. Accessed May 6, 2020.

[8] Statistisches Bundesamt . Bevölkerung und Erwerbstätigkeit.: Bevölkerung mit Migrationshintergrund ‐ Ergebnisse des Mikrozensus 2018. Fachserie 1 Reihe 2.2. 2019 https://www.destatis.de/DE/Themen/Gesellschaft‐Umwelt/Bevoelkerung/Migration‐Integration/Publikationen/Downloads‐Migration/migrationshintergrund‐2010220187004.pdf?__blob=publicationFile. Accessed May 6, 2020.

[9] Oldenburg C , Siefer A , Beermann B . Migration als Prädiktor für Belastung und Beanspruchung? In: Badura B, ed. Fehlzeiten‐Report 2010: Vielfalt managen : Gesundheit fördern—Potenziale nutzen : Zahlen, Daten, Analysen aus allen Branchen der Wirtschaft. Berlin, Heidelberg: Springer; 2010:141‐151.

[10] Brzoska P , Reiss K , Razum O . Arbeit, Migration und Gesundheit In: Badura B, ed. Fehlzeiten‐Report 2010: Vielfalt managen: Gesundheit fördern—Potenziale nutzen: Zahlen, Daten, Analysen aus allen Branchen der Wirtschaft. Berlin, Heidelberg: Springer; 2010:129‐139.

[11] Schröder CC , Dyck M , Breckenkamp J , Hasselhorn HM , Du Prel J‐B . Utilisation of rehabilitation services for non‐migrant and migrant groups of higher working age in Germany ‐ results of the lidA cohort study. BMC Health Serv Res. 2020;20(1):31 10.1186/s12913-019-4845-z 31924217 PMC6954536

[12] Brzoska P , Voigtländer S , Spallek J , Razum O . Arbeitsunfälle, Berufskrankheiten und Erwerbsminderung bei Menschen mit Migrationshintergrund In: Schott T, Razum O, eds. Migration und medizinische Rehabilitation. Weinheim: Beltz Juventa; 2013:49‐61.

[13] Brzoska P , Razum O . Erreichbarkeit und Ergebnisqualität rehabilitativer Versorgung bei Menschen mit Migrationshintergrund. Bundesgesundheitsblatt Gesundheitsforschung Gesundheitsschutz. 2015;58(6):553‐559. 10.1007/s00103-015-2144-3 25861042

[14] Eurofound . How your birthplace affects your workplace. Luxembourg: Publications Office of the European Union; 2019 https://www.Eurofound.europa.eu/sites/default/files/ef_publication/field_ef_document/ef19004en.pdf. Accessed May 6, 2020.

[15] Hasselhorn HM .Social inequality in the transition from work to retirement In: Theorell T, ed. Handbook of socioeconomic determinants of occupational health: From macro‐level to micro‐level evidence. Handbook Series in Occupational Health Sciences. 1st ed Berlin Heidelberg: Springer; 2020:1‐26. 10.1007/978-3-030-05031-3_32-1.

[16] Nilsson K , Hydbom AR , Rylander L . Factors influencing the decision to extend working life or retire. Scand J Work Environ Health. 2011;37(6):473‐480. 10.5271/sjweh.3181 21725583

[17] Ebener M . Die Erfassung der Motivation, erwerbstätig zu sein, in arbeitswissenschaftlichen Studien [Dissertation]. Wuppertal: Bergische Universität Wuppertal; 2018.

[18] Engstler H . Wie erfolgreich sind ältere Arbeitskräfte in der zeitlichen Umsetzung ihrer Ausstiegspläne? : Soziale Unterschiede der Übereinstimmung zwischen geplantem und realisiertem Alter der Erwerbsbeendigung. Z Gerontol Geriatr. 2019;52(Suppl 1):14‐24. 10.1007/s00391-018-1451-3 30267263

[19] Örestig J , Strandh M , Stattin M . A wish come true? A longitudinal analysis of the relationship between retirement preferences and the timing of retirement. Population Ageing. 2013;6(1):99‐118. 10.1007/s12062-012-9075-7

[20] Du Prel J‐B , Schrettenbrunner C , Hasselhorn HM . Vertikale und horizontale soziale Ungleichheit und Motivation zum vorzeitigen Erwerbsausstieg. Z Gerontol Geriatr. 2019;52(Suppl 1):3‐13. 10.1007/s00391-018-1450-4 30280240

[21] Hess M . Erwartetes und gewünschtes Renteneintrittsalter in Deutschland. Z Gerontol Geriatr. 2018;51(1):98‐104. 10.1007/s00391-016-1053-x 27125820

[22] Bélanger A , Sabourin P , Carrière Y . National Report Canada. Full report of the respective chapter In: Hasselhorn HM, Apt W, eds. Understanding employment participation of older workers: Creating a knowledge base for future labour market challenges [Research report]. Berlin: Federal Ministry of Labour and Social Affairs (BMAS) and Federal Institute for Occupational Safety and Health (BAuA); 2015.

[23] Deutsche Rentenversicherung Bund . Reha‐Bericht 2015: Die medizinische und berufliche Rehabilitation der Rentenversicherung im Licht der Statistik. Berlin: Deutsche Rentenversicherung Bund; 2015 https://www.deutsche‐rentenversicherung.de/SharedDocs/Downloads/DE/Statistiken‐und‐Berichte/Berichte/rehabericht_2015.pdf;jsessionid=2553F86938BC52C59D686E737E1A0A1F.delivery2‐7‐replication?__blob=publicationFile&v=1. Accessed May 6, 2020.

[24] Hasselhorn HM , Peter R , Rauch A , et al. Cohort profile: the lidA Cohort Study‐a German Cohort Study on Work, Age, Health and Work Participation. Int J Epidemiol. 2014;43(6):1736‐1749. 10.1093/ije/dyu021 24618186 PMC4276057

[25] Research Data Centre of the German Federal Employment Agency . Scientific Use File of lidA – leben in der Arbeit. https://fdz.iab.de/en/FDZ_Individual_Data/lidA.aspx. Accessed May 6, 2020.

[26] Schröder H , Kersting A , Gilberg R , Steinwede J . Methodenbericht zur Haupterhebung lidA‐leben in der Arbeit. FDZ‐Methodenreport 01/2013. Nürnberg: Forschungsdatenzentrum der Bundesagentur für Arbeit im Institut für Arbeitsmarkt‐ und Berufsforschung; 2013 http://doku.iab.de/fdz/reporte/2013/MR_01‐13.pdf. Accessed May 6, 2020.

[27] Steinwede J , Kleudgen M , Häring A , Schröder H . Methodenbericht zur Haupterhebung lidA‐leben in der Arbeit, 2. Welle. FDZ‐Methodenreport 07/2015. Nürnberg: Forschungsdatenzentrum der Bundesagentur für Arbeit im Institut für Arbeitsmarkt‐ und Berufsforschung; 2015 http://doku.iab.de/fdz/reporte/2015/MR_07‐15.pdf. Accessed May 6, 2020.

[28] Steinwede J , Ruiz Marcos J , Kleudgen M . Methodenbericht lidA Welle 3 [Unpublished technical report]. Nürnberg: Forschungsdatenzentrum der Bundesagentur für Arbeit im Institut für Arbeitsmarkt‐ und Berufsforschung; 2018.

[29] Schenk L , Bau A‐M , Borde T , et al. Mindestindikatorensatz zur Erfassung des Migrationsstatus. Empfehlungen für die epidemiologische Praxis. Bundesgesundheitsblatt Gesundheitsforschung Gesundheitsschutz. 2006;49(9):853‐860. 10.1007/s00103-006-0018-4 16927038

[30] Ahrens W , Bellach B , Jöckel K‐H . Messung soziodemographischer Merkmale in der Epidemiologie. München: MMV Medizin Verlag; 1998.

[31] OECD . What are equivalence scales. http://www.oecd.org/els/soc/OECD‐Note‐EquivalenceScales.pdf. Updated March 29, 2016. Accessed May 6, 2020.

[32] Lampert T , Kroll LE . Soziale Unterschiede in der Mortalität und Lebenserwartung. GBE kompakt. 2014;5(2). 10.17886/RKI-GBE-2016-017. Accessed May 6, 2020.

[33] Ware JE . How to score version 2 of the SF‐12 health survey: (with a supplement documenting version 1). Lincoln, R.I., Boston, MA: QualityMetric Inc; Health Assessment Lab; 2005.

[34] Nübling M , Andersen HH , Mühlbacher A . Entwicklung eines Verfahrens zur Berechnung der körperlichen und psychischen Summenskalen auf Basis der SOEP‐Version des SF 12 (Algorithmus). [Data Documentation]; 2006; 16. https://www.diw.de/documents/publikationen/73/diw_01.c.44987.de/diw_datadoc_2006‐016.pdf. Accessed May 6, 2020.

[35] Wahrendorf M , Dragano N , Siegrist J . Social position, work stress, and retirement intentions: A study with older employees from 11 European countries. Eur Sociol Rev. 2013;29(4):792‐802.

[36] Nübling M , Stößel U , Hasselhorn HM , Michaelis M , Hofmann F . Methoden zur Erfassung psychischer Belastungen Erprobung eines Messinstruments (COPSOQ). Bremerhaven: Wirtschaftsverlag NW; 2005.

[37] Siegrist J , Starke D , Chandola T , et al. The measurement of effort–reward imbalance at work: European comparisons. Soc Sci Med. 2004;58(8):1483‐1499. 10.1016/S0277-9536(03)00351-4 14759692

[38] Hasselhorn HM , Michaelis M , Kujath P . Die betriebsärztliche Betreuung von Erwerbstätigen – Ergebnisse der repräsentativen lidA‐Studie. ASU Arbeitsmed Sozialmed Umweltmed. 2020;03:198‐219.

[39] Brzoska P , Sauzet O , Breckenkamp J . Unobserved heterogeneity and the comparison of coefficients across nested logistic regression models: how to avoid comparing apples and oranges. Int J Public Health. 2017;62(4):517‐520. 10.1007/s00038-016-0918-5 27812725

[40] Hofäcker D . In line or at odds with active ageing policies? Exploring patterns of retirement preferences in Europe. Age Soc. 2015;35(7):1529‐1556. 10.1017/S0144686X1400035X

[41] Rinklake A , Buchholz S . Increasing inequalities in Germany: Older people’s employment lives and income conditions since the mid‐1980s In: Blossfeld H‐P, Buchholz S, Kurz K, eds. Aging populations, globalization and the labor market: Comparing late working life and retirement in modern societies. UK/Northampton, MA: Edward Elgar; 2011:35‐64.

[42] Roberts J , Rice N , Jones AM . Early retirement among men in britain and germany: How important is health? The Geneva Papers on Risk and Insurance ‐ Issues and Practice. 2010;35(4):644‐667. 10.1057/gpp.2010.24

[43] Wilson DM , Errasti‐Ibarrondo B , Low G , et al. Identifying contemporary early retirement factors and strategies to encourage and enable longer working lives: A scoping review. Int J Older People Nurs. 2020;15(3):e12313 10.1111/opn.12313 32166897

[44] Tucci I , Yildiz S . Das Alterseinkommen von Migrantinnen und Migranten: zur Erklärungskraft von Bildungs‐ und Erwerbsbiografien In: Baykara‐Krumme H, Schimany P, Motel‐Klingebiel A, eds. Viele Welten des Alterns. Wiesbaden: VS Verlag für Sozialwissenschaften; 2012:101‐123.

[45] Phillipson C . ‘Fuller’ or ‘extended’ working lives? Critical perspectives on changing transitions from work to retirement. Age Soc. 2019;39(3):629‐650. 10.1017/S0144686X18000016

